# Simulation Analysis on the Characteristics of Aerosol Particles to Inhibit the Infrared Radiation of Exhaust Plumes

**DOI:** 10.3390/ma17143505

**Published:** 2024-07-15

**Authors:** Wei Li, Yurou Wang, Lei Zhang, Baohai Gao, Mingjian He

**Affiliations:** 1 School of Energy Science and Engineering, Harbin Institute of Technology, Harbin 150001, China; liwei1988629@126.com (W.L.); 21s002101@stu.hit.edu.cn (Y.W.); hemingjian@hit.edu.cn (M.H.); 2 Shenyang Aircraft Design and Research Institute, Aviation Industry Corporation of China, Shenyang 110035, China; zhl962@163.com; 3 Key Laboratory of Aerospace Thermophysics, Ministry of Industry and Information Technology, Harbin 150001, China; 4 National Key Laboratory of Electromagnetic Information Control and Effects, Aviation Industry Corporation of China, Shenyang 110035, China

**Keywords:** aerosol particle, exhaust plume, infrared radiation characteristics, radiation intensity

## Abstract

Aerosol infrared stealth technology is a highly effective method to reduce the intensity of infrared radiation by releasing aerosol particles around the hot exhaust plume. This paper uses a Computational Fluid Dynamics (CFD) two-phase flow model to simulate the exhaust plume fields of three kinds of engine nozzles containing aerosol particles. The Planck-weighted narrow spectral band gas model and the Reverse Monte Carlo method are used for infrared radiation transfer calculations to analyze the influencing factors and laws for the suppression of the infrared radiation properties of exhaust plumes by four typical aerosol particles. The simulation calculation results show that the radiation suppression efficiency of aerosol particles on the exhaust plume reaches its maximum value at a detection angle (*ϕ*) of 0° and decreases with increasing *ϕ*, reaching its minimum value at 90°. Reducing the aerosol particle size and increasing the aerosol mass flux can enhance the suppression effect. In the exhaust plume studied in this paper, the radiation suppression effect is best when the particle size is 1 μm and the mass flux is 0.08 kg/s. In addition, the inhibition of aerosol particles varies among different materials, with graphite having the best inhibition effect, followed by H_2_O, MgO, and SiO_2_. Solid particles will increase the radiation intensity and change the spectral radiation characteristics of the exhaust plume at detection angles close to the vertical nozzle axis due to the scattering effect. Finally, this paper analyzed the suppression effects of three standard nozzle configurations under the same aerosol particle condition and found that the S-bend nozzle provides better suppression.

## 1. Introduction

Due to the growing perfection of infrared (IR) radiation theory, IR detectors, and signal processing technology, the development of IR imaging technology has become a key means in the application of target identification and detection for new-generation military systems since the Second World War [1,2,3]. Infrared-guided weapons use IR imaging technology and IR sensors to lock onto the target and ensure the precision of the strike, featuring high precision, a strong anti-jamming capability, long attack distance, and good concealment and are not easily interfered with by radar systems [4]. Furthermore, infrared-guided weapons can be used at night or in low-light environments and are not easily detected by the enemy. Simultaneously, integrating IR guidance technology and other guidance technologies to form composite guided weapons enhances their combat power on the battlefield, increasing their range and accuracy. This improvement is significant and objective, as it is based on technological advancements [5]. The aircraft’s survivability is seriously threatened by the IR thermal radiation emitted from the engine exhaust system [6]. According to relevant statistics, 70–80% of damaged aircrafts were destroyed by IR thermal radiation-guided missiles in recent local wars [7].

Military targets face great dangers and challenges under increasingly harsh living conditions caused by air forces. Major military powers are developing advanced anti-infrared detection technology to avoid detection by IR detectors and increase the survival of military weapons [8,9,10]. IR stealth technology is one such development, which reduces the intensity of IR radiation in the detection bands of fighter jets, giving them a more vital ability to remain undetected on the battlefield. The conventional methods to suppress the IR radiation are cooling down the nozzle temperature, coating lower-emissivity materials, or installing shelters to block IR radiation [11,12,13]. Aerosol infrared stealth technology is a significant method for achieving IR stealth by studying the infrared radiation emitted from high-temperature tail spray flames in two atmospheric window bands: 3–5 μm and 8–14 μm. Its working principle follows: Under turbulence, atmospheric buoyancy, and other factors, solid aerosol particles, such as graphite and silicon dioxide, of micron or nanometer scale are suspended and surrounded by the tail nozzle flame. At the same time, the tail nozzle around the formation of a solid particle layer of IR radiation also has a good shielding effect, significantly reducing the intensity of IR radiation to achieve the purpose of IR stealth [14,15,16,17]. Compared with other stealth technologies, the aerosol IR stealth technology has the advantage of not changing the aerodynamic characteristics and having less aerodynamic thrust loss.

As for the infrared extinction characteristics of discrete particles, Shi [18] calculated the infrared extinction properties of brass aerosols, assuming a lognormal distribution function for the particle size and a spherical particle shape, and the results of Lambert–Beer law and generalized theory are also given and compared. Yurkin [19] analyzed the smoke screen transmittances of aerosol particles of different shapes in relation to the particle number density, smoke screen thickness, and angle of incidence. Wang [20] found that the shape of the particles influences the transmittance rate of the smoke screen, the number density of the particles, the angle of incidence of the incident light, and the thickness of the smoke screen, showing that the more irregular the shape of the particles, the lower the transmittance. Considering the results of the above research, the properties of aerosol particles, including their material, size, and mass flux, need to be discussed in the context of this paper. The impact of the spray-in mode of aerosol particles on the radiation suppression effect was studied by the research team of Nanjing University of Aeronautics and Astronautics [21,22] by using a combination of CFD numerical simulation and ground experiments to conduct an in-depth and systematic study on the influence of various parameters on the particle distribution and infrared radiation characteristics of thermal jets and the design of the injection system. Myong [23] considered water droplets and carbon particles with diameters of 5 μm and 10 μm to analyze the shielding effects of different particle sizes, material types, and distribution patterns. Gao [24] analyzed the influence of various parameters, including the particle injection flow rate, injection velocity, injection angle, diameter, and outflow Mach number, on the infrared radiation characteristics of the exhaust system after particle injection. Sun [25] found that the baffle installed at the end of the nozzle had a significant effect on the diffusion of aerosol particles in the shear flow, with the linear baffle structure being superior to the curved one, and the uniformity of the particle distribution improving with the increase in the number of nozzles. Several typical studies were selected to summarize their selected aerosol particle materials, diameters, and mass flow rates. These data provide reference for the parameter selection in this paper. As shown in Table 1, the particle size of aerosol particles used for IR suppression is usually in the range of 0.1 µm–100 µm. Most of the materials are silicon dioxide (SiO_2_), graphite (C), etc. The mass flux is also related to the size of the computational model, and the aerosol mass flux chosen varies from one to another, usually between 0.02 kg/s and 1 kg/s.

Most of the studies on aerosol IR suppression in the existing literature are based on a single model with a certain nozzle shape. In addition, the material, particle size, and mass flux of the aerosol are not included in the overall considerations, and atmospheric attenuation and environmental radiation are not taken into account.

The purpose of this study is to obtain the influencing factors and laws for the suppression of the infrared radiation properties of nozzle exhaust plumes. The flow field of the nozzle exhaust plume is first simulated using numerical computation. Then, an automatic preprocessing model of the flow field was constructed to overcome the mismatch between the CFD grid and the radiation transport grid so that the flow field information could be identified quickly. The absorption coefficients of the tail gas spectrum as well as the absorption and scattering coefficients of the particles were calculated using a high-spectral-resolution Planck-weighted narrow spectral band gas model and Mie theory. Finally, the concept of the radiation distribution factor (RDF) is introduced, and the Reverse Monte Carlo (RMC) method, which is suitable for calculating complex radiation models, is used so as to calculate the infrared intensity distributions of nozzle exhaust plumes containing aerosol particles. The study investigated the impact of particle size, mass flux, and four aerosol materials, including graphite (C), magnesium oxide (MgO), silicon dioxide (SiO_2_), and the mist of water (H_2_O), on the suppression of IR radiation to find the optimal suppression conditions. This paper also investigated the effects of three different nozzle shapes (convergent nozzles, S-bend nozzles, and convergent-divergent nozzles) on the suppression efficiency of IR radiation under the same aerosol conditions. This work is of great academic and applied significance for enriching the theory of aerosol IR suppression and developing effective control strategies for aerosol distribution patterns.

## 2. Numerical Methods

### 2.1. Computational Model and Setup

**Figure 1:**
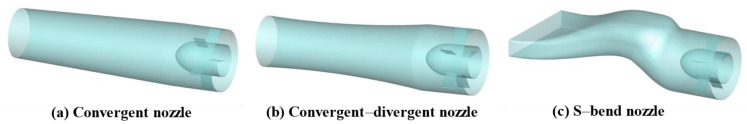
Physical models of three types of engine tail nozzles.

**Figure 2:**
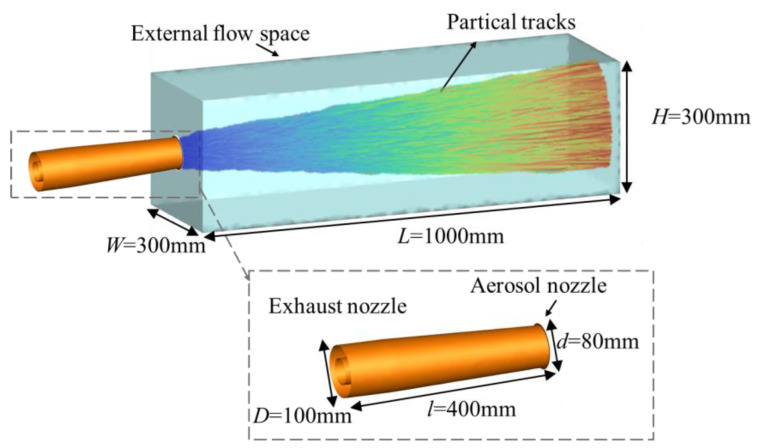
The computational model of convergent nozzle.

In this paper, ANSYS SCDM and FLUENT MESHING software are employed to construct and mesh the computational model of the engine tail nozzles. Furthermore, a series of flow field data, including the temperature and pressure field of the tail nozzle containing aerosol particles, the concentration distribution of the components, and discrete phase (aerosol) particles, are calculated in the CFD solver of Ansys Fluent 17.0 (2022). The flow field nozzle takes three typical physical models of an engine tail nozzle: convergent nozzle, S-bend nozzle, and convergent–divergent nozzle, as shown in Figure 1. In order to reduce the calculation cost, the computational model was reduced to 1/13 of the original geometry. The internal structure of the nozzle was simplified, retaining the outer wall of the nozzle, the center cone of the nozzle, and the support plate, and all solid materials of the nozzle were aluminum (Al). Figure 2 displays the computational model containing the aircraft exhaust convergent nozzle, the aerosol nozzle, and the external flow domain. The exhaust nozzle has an inlet diameter of 100 mm, an outlet diameter of 80 mm, and a length of 400 mm. The aerosol jet injection area is located near the outer culvert of the nozzle outlet, with a distance of 1 mm from the center of the nozzle to the upper wall of the exhaust nozzle. The external flow domain is defined as a cuboid, with dimensions of 1000 mm in length, 300 mm in width, and 300 mm in height. The inlet and outlet diameters of the convergent–divergent nozzle are the same as those of the convergent nozzle. The outlet shape of the S-bend nozzle is rectangular, with a length of 180 mm and a width of 28 mm. The outlet area of the three types of nozzles is consistent. All three nozzles have the same axial length of 400 mm.

**Figure 3:**
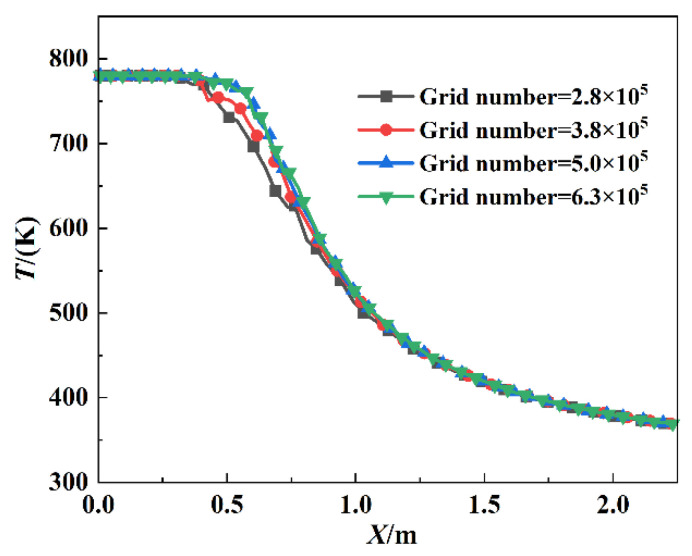
The grid independence test.

The flow field model with a convergent nozzle was divided into 500 thousand tetrahedral grids, with the fine mesh in the hot exhaust jet and the aerosol jet and coarse mesh in the external airflow space. The gas temperature distribution along the centerline was evaluated considering four different numbers of mesh elements, and the results are presented in Figure 3. It is obvious that the temperature change can be almost negligible after the grid cells are increased to 500,000. To reduce the computational time and cost without compromising the accuracy, the whole study was conducted considering 500,000 mesh elements within the computational domain. The other two nozzle models are identical and will not be repeated.

The SST k-omega model in ANSYS Fluent is suitable for various applications, including calculating the airfoil shape and supersonic speed and designing turbulent viscosity. Therefore, this paper employs the SST k-omega model to simulate the turbulence model, and the component transport model is chosen. The discrete Lagrangian model is appropriate for particle loading below 10%. The computational model involves a two-phase coupled flow of solid (aerosol) fluid, which satisfies this condition, so the discrete phase model (DPM) is used to track all aerosol particles in the flow.

When simulating a two-phase flow containing discrete particles, it is essential to consider the Saffman force to simulate the motion carried by the airflow. Additionally, a temperature gradient is inevitable due to the significant difference between the temperature of the tail-jet flame and the ambient temperature. Therefore, the thermophoretic force must also be taken into account.

A crucial aspect of discrete phase modeling is determining the spray pattern of aerosol particles. This paper employs the ‘cone’ injection mode to improve the simulation of particle motion in the air. The initial diameter of the ‘cone’ and the expansion angle of the ‘cone’ are two crucial injection parameters. The cone’s initial diameter is determined by the exit diameter of the aerosol nozzle and is set to 0.001 m. Furthermore, the expansion angle greatly influences the distribution characteristics of the aerosol particles around the tail flame. The nozzles for spraying aerosol particles are of the same diameter and have a half-expansion angle of 6°. The hot exhaust plume is set as a pressure inlet; the pressure is 129,696 Pa, and the temperature is 780 K. The gas components contain CO_2_, H_2_O, N_2_, and O_2_, with a component mole concentration of 0.03 for CO_2_ and H_2_O. When the air temperature is 300 K in ground condition, the pressure of the exhaust plume and the air pressure are set as 1 atm. The simulations are performed using the commercial software ANSYS Fluent.

### 2.2. Flow Field Pretreatment

**Figure 4:**
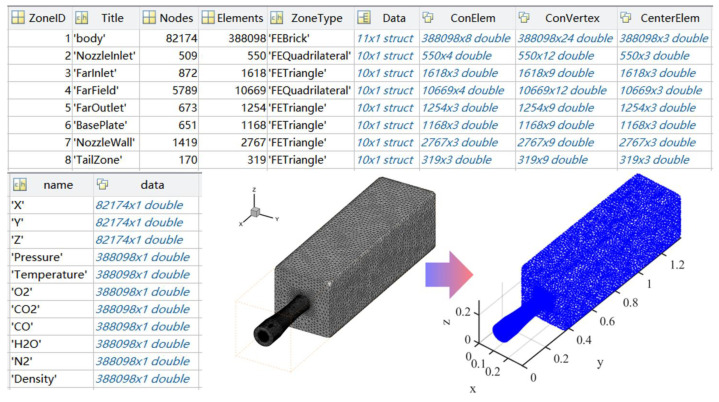
Flow field preprocessing and model visualization.

To identify and read the flow field data files, the traditional preprocessing method for flow fields derives the grid nodes’ spatial coordinates and the flow field’s corresponding parameters, such as the temperature, pressure, and component concentration. The method then interpolates the inverse of the distance of the flow field grid, adjusts the interpolated grid sparsity, and verifies the reconstructed mesh quality by comparing it with the original flow field. However, generating structural grids for complex geometries can be difficult, which limits the application of traditional preprocessing methods to simple and regular nozzle geometries, causing problems due to mismatches between the radiation and flow grids. This paper presents an automated software for preprocessing flow field data in CFD simulations. The software can identify unstructured grid data files and export the surface and volume cell names, grid types, grid correlations, grid node spatial coordinates, and corresponding flow field parameters such as the temperature, pressure, and component concentration, shown in Figure 4, and the system can automatically adjust the orientation of the tail flames and display the flow field grid cell by cell.

### 2.3. Mathematical Model

#### 2.3.1. Gas Radiation Characteristics

The Line-by-Line (LBL) method is considered the most accurate currently available method and can serve as a reference for other methods. It is necessary to provide each spectral line’s specific spectral line characteristic coefficients. The absorption region of a gas exhibits overlapping spectral lines. For the same gas, its spectral absorption coefficient $\kappa_{\eta }$ at wave number η equals the sum of the line absorption coefficients $\kappa_{\eta i}$ of the individual mutually overlapping spectral lines at wave number η [26].

The spectral absorption coefficient equation can be written as

(1) $$\kappa_{\eta }=\sum_{i}\kappa_{\eta i}=\sum_{i}S_{i}F(\eta -\eta_{0i})$$

where $\kappa_{\eta i}$ is the absorption coefficient of the *i*th spectral line at the wave number; $F(\eta -\eta_{0i})$ is the line function of the *i*th spectral line; $\eta_{0i}$ is the wave number at the center of the *i*th spectral line in the computational domain; and $S_{i}$ is the integrated intensity of the *i*th spectral line.

The LBL method cannot satisfy the requirement of calculation time when calculating the infrared radiation characteristics of a certain band, while the narrow spectral band model can satisfy the requirement of calculation accuracy and calculation time at the same time. In all narrow spectral band models, it is basically assumed that the Planck function within the spectral band is invariant, and this approach will produce some error. Considering the Planck function weighting in the narrow spectral bands, the Planck-weighted spectral absorption coefficient in the narrow spectral interval (η−Δη/2,η+Δη/2) at any central wave number η can be expressed as

(2) $$\kappa_{P,\;k}=\frac{\int_{\eta_{1}}^{\eta_{2}}\kappa_{\eta }I_{b\eta }\;d\eta }{\int_{\eta_{1}}^{\eta_{2}}I_{b\eta }\;d\eta }$$

where subscript *k* represents the *k*-th narrow spectral band, $\eta_{1}\sim \eta_{2}$ is the corresponding narrow spectral band interval, and the interval between each narrow band is 25 cm^−1^ in this paper. *η* is the spectral wavenumber (cm^−1^), which is the reciprocal of the wavelength *λ*; $I_{b\eta }$ is the blackbody radiation intensity at the center of the narrow spectral line; the subscript ‘P’ expresses the weighting of the Planck function.

Since the narrow spectral band absorption coefficient is the sum of the contributions of the individual spectral lines, Equation (2) can be changed into

(3) $$\kappa_{P,\;k}=\frac{\sum_{i}\int_{\eta_{1}}^{\eta_{2}}\kappa_{i\eta }I_{b\eta }\;d\eta }{\int_{\eta_{1}}^{\eta_{2}}I_{b\eta }\;d\eta }=\frac{\sum_{i}I_{b\eta_{i,\;k}}S_{i,\;k}}{\int_{\eta_{1}}^{\eta_{2}}I_{b\eta }d\eta }$$

In this paper, the Planck-weighted narrow spectral band radiative properties database for gases is based on the latest HITEMP2020 database [27]. The high-resolution spectral absorption coefficients (0.01 cm^−1^) of the gases were firstly calculated using the Line-by-Line method, and then, their Planck-weighted narrow spectral band absorption coefficients were calculated using Equation (3). Two gas molecules (H_2_O and CO_2_) were calculated. The gas pressure ranges from 2000 Pa to 1,500,000 Pa (equally divided into 100 parts), the temperature ranges from 200 to 2500 K (equally divided into 100 parts), the component concentration ranges include [0, 10^−4^, 10^−3^, 0.01, 0.02, 0.05, 0.1, 0.4, 0.7, 1.0], and the spectral ranges from 150 to 9300 cm^−1^ with a spectral resolution of 5 cm^−1^. Spectral absorption coefficients for other states can be calculated using linear interpolation.

#### 2.3.2. Particle Radiation Characteristics

A single particle’s radiation properties can be transformed into a beam of plane electromagnetic waves projected onto a particle with specific size and optical parameters according to electromagnetism principles. This transformation is described by the solution of Maxwell’s system of equations, known as the Mie theory, which is a particular application of electromagnetism in the radiation of particles [28,29]. The Mie scattering formula is based on the solution of Maxwell’s system of equations for a plane electromagnetic wave interacting with homogeneous spherical particles in the far field [30].

The final problem of the Mie scattering formula is to solve the scattering coefficients $a_{n}$ and $b_{n}$ as well as the scattering angle functions $\pi_{n}$ and $\tau_{n}$. The scattering coefficients $a_{n}$ and $b_{n}$ are ratios of complex functions with complex independent variables, which are prone to unstable solutions [31,32]. Since the 1960s, computer programs have greatly improved the accuracy of calculation results and expanded the range of applicable parameters [33,34]. The Mie theory-based solution method provides accurate solutions for smooth spherical particles in the Mie scattering region and is one of the most important methods in particle scattering.

The simplified treatment using Mie theory is due to the difficulty in determining the carbon black particles in the aero-engine tail flame. When a plane electromagnetic wave of wavelength *a* is projected onto a single isotropic homogeneous spherical particle, using the Legendre polynomials, the functions $\pi_{n}$ and $\tau_{n}$ form an orthogonal set of functions, and the attenuation factor $Q_{\mathrm{ext}}$, scattering factor $Q_{\mathrm{sca}}$, and absorption factor $Q_{\mathrm{abs}}$ of the particle can be expressed using the Lorenz–Mie electromagnetic theory as [28]:

(4) $$Q_{\mathrm{ext}}=\frac{2}{x^{2}}Re[\sum_{n=1}^{\infty }(2n+1)(a_{n}+b_{n})]$$

(5) $$Q_{\mathrm{sca}}=\frac{2}{x^{2}}\sum_{n=1}^{\infty }(2n+1)(|a_{n}{|}^{2}+|b_{n}{|}^{2})$$

(6) $$Q_{\mathrm{abs}}=Q_{\mathrm{ext}}-Q_{\mathrm{sca}}$$

where *Re* means to take the real part of the complex number, x=πD/λ is the scale parameter, *D* is the particle size, *λ* is the wavelength, and $a_{n}$ and $b_{n}$ are the Mie scattering coefficients, respectively.

(7) $$a_{n}=\frac{\psi \prime_{n}(mx)\psi_{n}(x)-m\psi_{n}(mx)\psi \prime_{n}(x)}{\psi \prime_{n}(mx)\xi_{n}(x)-m\psi_{n}(mx)\xi \prime_{n}(x)}$$

(8) $$b_{n}=\frac{m\psi \prime_{n}(mx)\psi_{n}(x)-\psi_{n}(mx)\psi \prime_{n}(x)}{m\psi \prime_{n}(mx)\xi_{n}(x)-\psi_{n}(mx)\xi \prime_{n}(x)}$$

Here, m=n−ik is the particle complex refractive index, where the real part *n* responds to the scattering properties of the particle, and the imaginary part *k* responds to the absorption properties of the particle. $\xi_{n}=\psi_{n}+i\chi_{n}$, $\psi_{n}(x)$, and $\chi_{n}(x)$ are the Ricatti–Bessel functions, satisfying the following recurrence relation:

(9) $$\begin{array}{l} \psi_{n+1}(x)=\frac{2n+1}{x}\psi_{n}(x)-\psi_{n-1}(x)\\ \chi_{n+1}(x)=\frac{2n+1}{x}\chi_{n}(x)-\chi_{n-1}(x) \end{array}$$

(10) $$\begin{array}{c} \psi_{-1}(x)=\cos x\;\;\;\psi_{0}(x)=\sin x\\ \chi_{-1}(x)=-\sin x\;\;\;\chi_{0}(x)=\cos x \end{array}$$

The amounts of particle scattering and absorption are expressed in terms of the scattering cross-section $C_{sca}$ and the absorption cross-section $C_{abs}$

(11) $$C_{abs}=Q_{abs}\cdot \pi \cdot {\left(D/2\right)}^{2}$$

(12) $$C_{sca}=Q_{sca}\cdot \pi \cdot {\left(D/2\right)}^{2}$$

(13) $$C_{ext}=Q_{ext}\cdot \pi \cdot {\left(D/2\right)}^{2}$$

If the particle coefficient density *N*(*D*) is known, the absorption scattering coefficient of the particle system can be found using the following equation:

(14) $$K_{abs}=\int_{0}^{\infty }C_{abs}\left(D\right)N\left(D\right)dD$$

(15) $$K_{sca}=\int_{0}^{\infty }C_{sca}\left(D\right)N\left(D\right)dD$$

(16) $$K_{e}=\int_{0}^{\infty }C_{ext}\left(D\right)N\left(D\right)dD$$

#### 2.3.3. Radiative Transfer Calculations

The current methods for solving the numerical problems of radiative heat transfer can be broadly classified into two main categories: (1) differential methods, which include the finite element method [35] and finite volume method [36] and (2) ray methods, which encompass the non-sequential ray-tracing method [37] and Monte Carlo method (MCM) [38]. Among these methods, finite element, finite volume, and ray-tracing methods are primarily employed to address the radiative heat transfer problem, which pertains to the distribution of radiant energy in space. In this paper, we require a high-directional-resolution radiant intensity solution method that can be employed to calculate the radiant intensity at any location along any orientation. The Reverse Monte Carlo method (RMC) considers all factors in radiative heat transfer, including emission, absorption, reflection, and scattering, during the ray-tracing process [39]. It is not dependent on geometrical shapes, volumes, or angular discretization and requires no approximations. Compared to the MCM method, RMC is more efficient in dealing with problems such as the calculation of infrared radiation characteristics of exhaust system targets that only care about the energy received by the detector in a certain direction.

**Figure 5:**
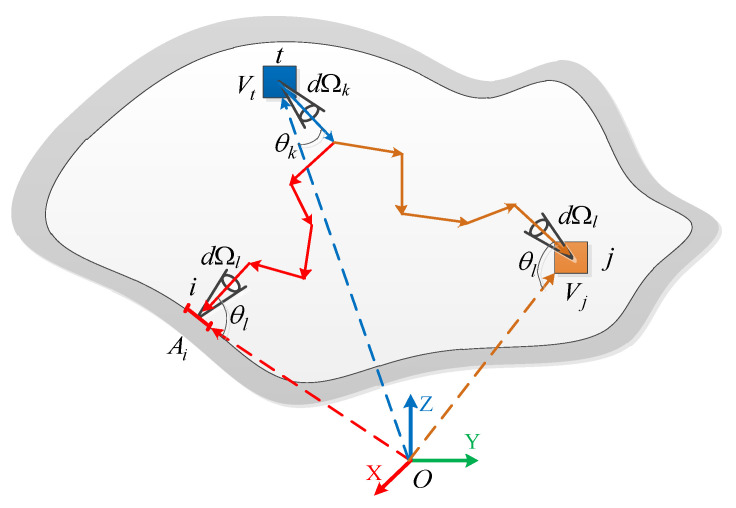
RMC ray-tracing modeling in multidimensional participatory media [36].

Thus, RMC can precisely calculate the directional radiation intensity at any point and is suitable for radiative systems with intricate geometries, particularly for computing radiative transfer in complex gas–solid two-phase media. The concept of the radiation distribution factor (RDF) is introduced to decompose the probabilistic simulation and iterative temperature calculation in the traditional RMC method. The RDF records the distribution of radiant energy in the participating medium and establishes the connection between the directional radiation intensity and the RDF and medium temperature [40,41,42]. The RMC method establishes a model for the radiative energy transfer path, as shown in Figure 5.

The whole control body is divided into *N* cells (including *N_v_* body cells and *N_s_* surface cells). The radiant energy emitted by the surface element *i* (or volume element *j*) and absorbed by the element *t* along the $\theta_{k}$ direction within the microelement stereo angle $d\Omega_{k}$ [36] can be expressed as follows:

(17) $$Q_{i}^{t,k}=\sum_{l=1}^{M}Q_{i,l}^{t,k}=\pi \varepsilon_{i}A_{i}I_{b}(T_{i})RD_{i}^{t,k}$$

(18) $$Q_{j}^{t,k}=\sum_{l=1}^{M}Q_{j,l}^{t,k}=4\pi \kappa_{j}V_{j}I_{b}(T_{j})RD_{j}^{t,k}$$

where the directional radiation distribution factor $RD_{i}^{t,k}$($RD_{j}^{t,k}$) denotes the fraction of radiant energy emitted by the surface cell *i* (or the volume cell *j*) and absorbed by the cell *t* along the angle $\theta_{k}$ within the micrometric stereoscopic angle $d\Omega_{k}$. *M* is the number of angular divisions of the 4π space.

Therefore, the energy absorbed by cell *t* in the micro elementary stereo angle $d\Omega_{k}$ along the direction $\theta_{k}$ can be expressed [36] as

(19) $$Q^{t,k}=\sum_{i=1}^{Ns}\pi \varepsilon_{i}A_{i}I_{b}(T_{i})RD_{i}^{t,k}+\sum_{j=1}^{N_{V}}4\pi \kappa_{j}V_{j}I_{b}(T_{j})RD_{j}^{t,k}$$

The intensity of directional radiation $I^{tk}$ at position *t* is independent of whether it is a surface or volume cell. It is only related to the medium’s temperature distribution and radiation distribution factor. Therefore, the radiation intensity in any direction at any position can be expressed using the following uniform formula [36]:

(20) $$I^{tk}=\sum_{n=1}^{N=N_{S}+N_{V}}RD_{t,k}^{n}\left(\frac{\sigma T_{n}^{4}}{\pi }\right)$$

#### 2.3.4. Detection Models

**Figure 6:**
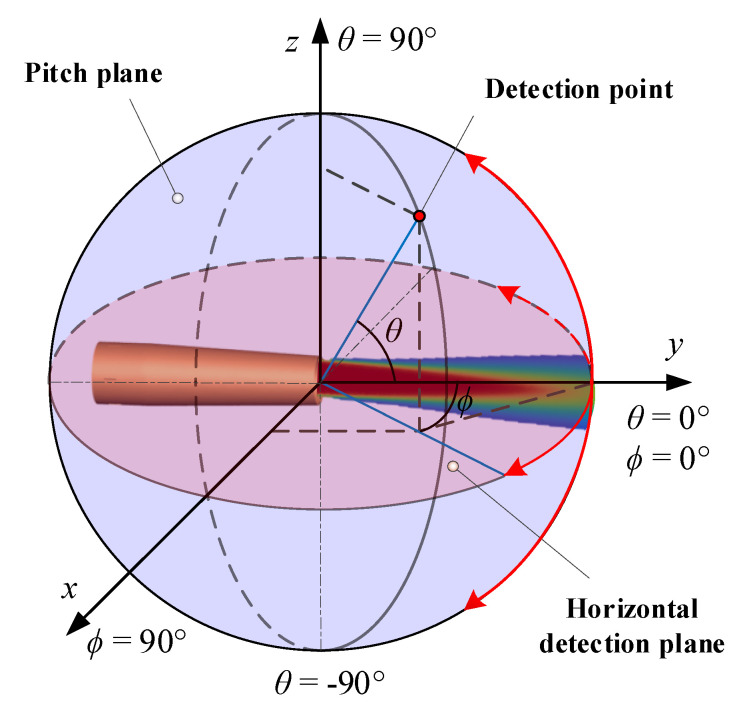
Definition of infrared detection angle.

The virtual infrared camera used for the simulation of exhaust plume radiation intensity is set to detect the spectral band of medium wave (3–5 μm). The simulation scenario adopts the ground-based detection mode, the altitude of the infrared camera and the tail flame of the aircraft are both 0 m, and the imaging detection distance is 15 m. The infrared detection angle is defined by the pitch and azimuth angles, as shown in Figure 6. The detection pitch angle is 0° for the engineering test, considering that the convergent nozzle has axisymmetric characteristics. The detection azimuth angles are selected as 0°, 5°, 10°, 15°, 20°, 30°, 45°, 60°, 75°, and 90°. The boundaries are diffusely reflected.

## 3. Results and Discussion

This section systematically discusses the effects of aerosol particle size, mass flux, material, and nozzle shape on the suppression efficiency of infrared radiation. The aim is to identify the characteristics of the aerosol effect on the suppression of infrared radiation from the tail-jet flame.

### 3.1. Model Validation of Numerical Methods for Infrared Radiation

**Figure 7:**
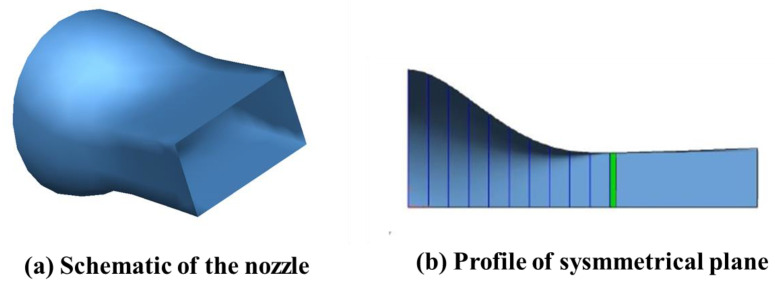
The schematic diagram of the validated model: (**a**) similar model constructed in this paper; (**b**) symmetric plane of circle-rectangular nozzle from reference [43].

**Figure 8:**
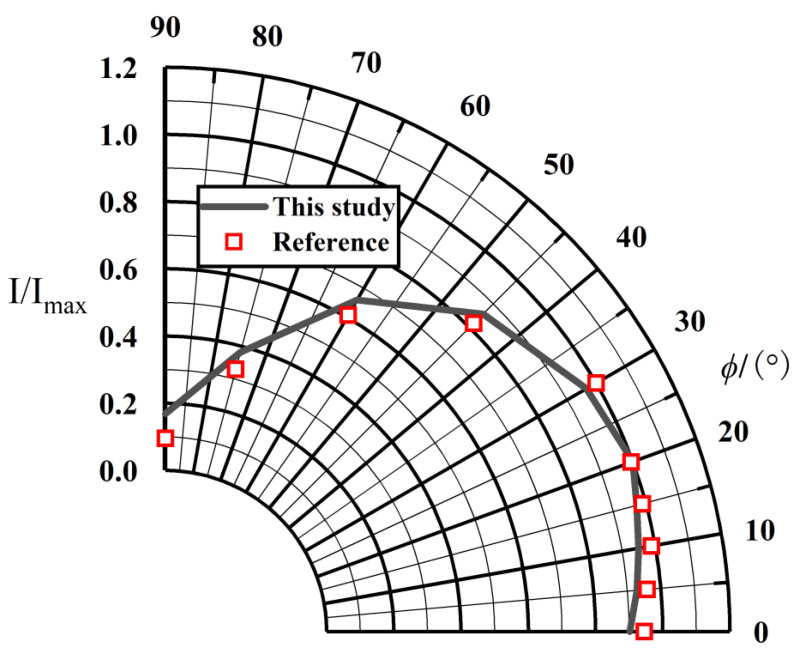
The comparison between the reference values [43] and the calculated values.

To verify the correctness of the model in this paper, the results of the simulation of the infrared radiation characteristics of a Circle-to-Rectangular C-D nozzle carried out by Cui et al. [43] were analyzed. The nozzle model A from the reference was selected as the numerical validation example. The same tail-jet model was built at a 1:1 scale, as shown in Figure 7. The IR transfer calculations were carried out by adopting the methodology studied in this paper, and the IR intensity values of the tail jet were obtained. The comparison between the reference values and the calculated values is given in Figure 8. The curve in the figure shows that the calculated results of this paper are in good agreement with the reference values, which shows the correctness of the infrared radiative transfer calculation model in this paper.

### 3.2. Influence of Aerosol Particle Size on Infrared Radiation Intensity

The influence of particle size on the infrared radiation characteristics of thermal jets is mainly reflected in the following aspects:

(1) When the mass concentration is fixed, a change in the particle size directly affects the number of particles in the flow field, affecting the calculation results of the transmittance.

(2) A change in the particle size at a certain incident wavelength alters the scattering phase function and the particle extinction efficiency factor *Q_ext_*.

(3) Changes in the particle size affect the weights of individual particles, which, in turn, affects the diffusion process in the jet to a certain degree. Additionally, the weight of particles in the jet changes due to energy consumption. Simultaneously, the diffusion of the jet itself is influenced to varying degrees by the consumption of fluid pulsation energy.

**Figure 9:**
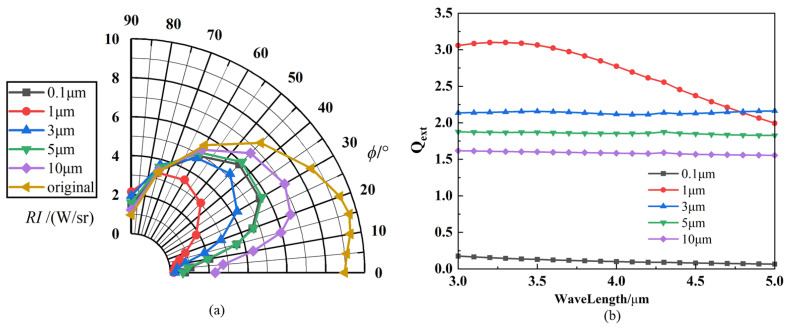
IR characteristics with different particle sizes (graphite): (**a**) IR intensity of nozzle exhaust plume; (**b**) extinction efficiency factor (*Q_ex_*_t_) of particles.

Figure 9a illustrates the directional infrared radiation intensity distribution of nozzle exhaust plume sprayed with five different sizes of particles and compares it with the original model without aerosol particle injection. In the subsequent results of this paper, the ‘original’ represents no aerosol sprayed in the nozzle. The angular distribution of infrared radiation intensity in the exhaust system under different particle sizes is significantly different. It is not the case that the larger the particle size, the more influential the suppression of infrared radiation is. Still, instead, an appropriate particle size allows for the best suppression of infrared radiation at the same particle injection flow rate. As shown in Figure 9b, when the particle size is small (*D* < 1 µm), the extinction efficiency factor (*Q_ext_*) of the particles is low or even tends to zero, which is an unfavorable factor for scattering. However, for the same mass flux, the smaller the particle size, the larger the particle concentration, which is favorable for the attenuation and absorption of the particle system. Consequently, particles with a diameter of 0.1 µm also exhibit an infrared suppression effect. As the particle size increases (*D* > 1 µm), the extinction efficiency coefficient decreases. Furthermore, an increase in the particle size leads to a decrease in the number of particles in the flow field, which results in a decrease in IR suppression with an increasing particle size.

**Figure 10:**
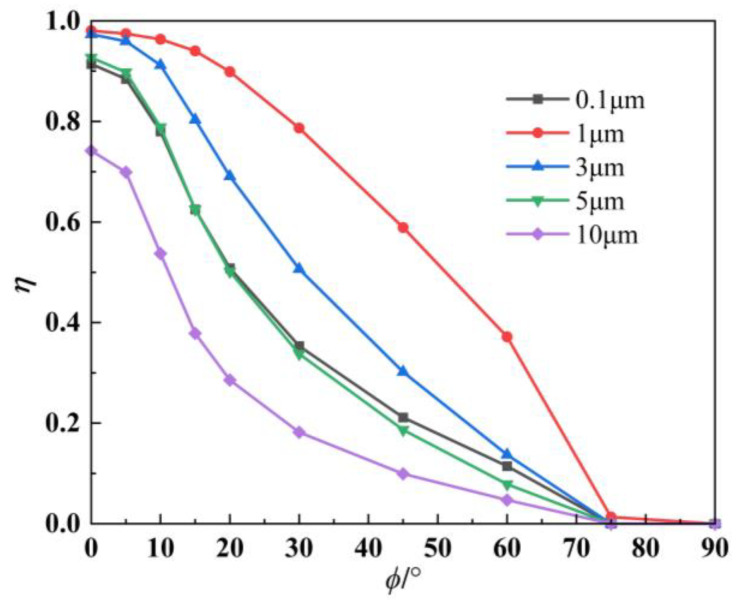
IR suppression rate under different aerosol particle sizes.

The IR radiation intensity suppression efficiency curves are illustrated in Figure 10 to visualize the IR suppression effect for different aerosol particle sizes. Various calculation models reach the maximum value of suppression efficiency at a detection angle (*ϕ*) of 0°, because at this angle, the absorption and scattering effects are powerful, resulting in a significant attenuation of IR radiation and a small final IR radiation intensity received by the detector. However, as the detection angle increases, the effective projected area of the observed tail jet also increases, resulting in a corresponding increase in the intensity of the IR radiation. Additionally, it can be observed from the figure that the rate of IR suppression generally decreases with an increasing *ϕ*, reaching a minimum at 90°. The aerosol nozzles are spaced so that the particles need to move axially for some distance before they converge in the circumferential direction to form an encapsulated particle layer outside the thermal jet. On the other hand, as the angle increases, the thickness of the particle layer decreases, leading to a faster decrease in the inhibition rate.

**Figure 11:**
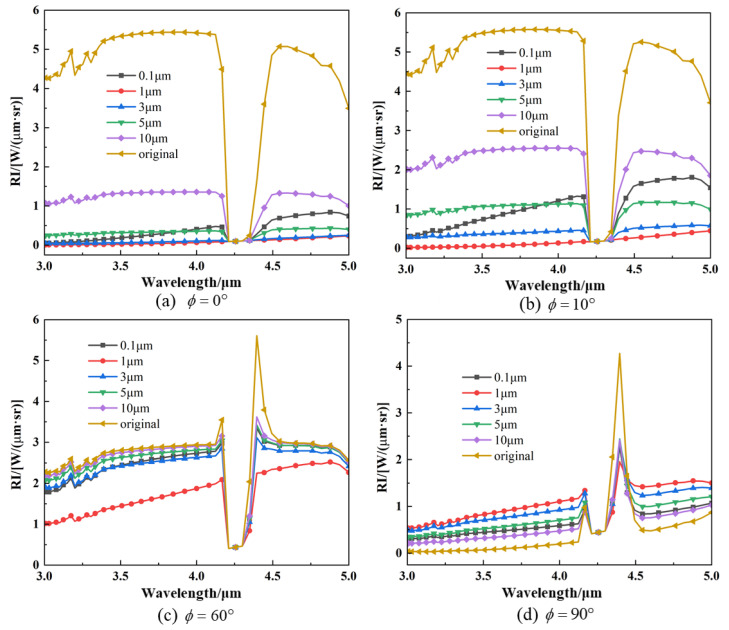
Spectral IR intensity of nozzle exhaust plume with different particle sizes: (**a**) detection angle of *ϕ* = 0°; (**b**) detection angle of *ϕ* = 10°; (**c**) detection angle of *ϕ* = 60°; (**d**) detection angle of *ϕ* = 90°.

Figure 11 displays the spectral radiation intensity distribution curves at different detection angles. It can be seen that the particle injection can reduce the intensity of the target infrared radiation received by the detector at most of the detection angles. The injection of particles causes the radiation intensity to be non-spectral in a certain band range (3 μm–4.2 μm and 4.4 μm–5.0 μm) due to the scattering of the target radiant energy by the particle-containing medium system, allowing the detector to pick up radiant energy from solid surface elements at large detection angles.

**Figure 12:**
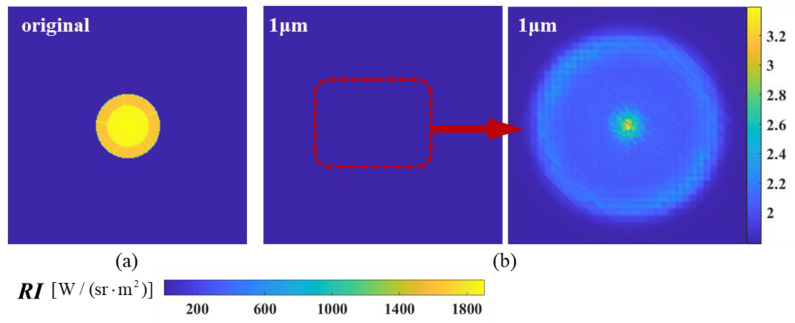
The near-field radiation imaging of the nozzle exhaust system at a detection angle of *ϕ* = 0°: (**a**) original model without aerosol spraying; (**b**) the nozzle sprays aerosol particles with a diameter of 1 µm.

**Figure 13:**
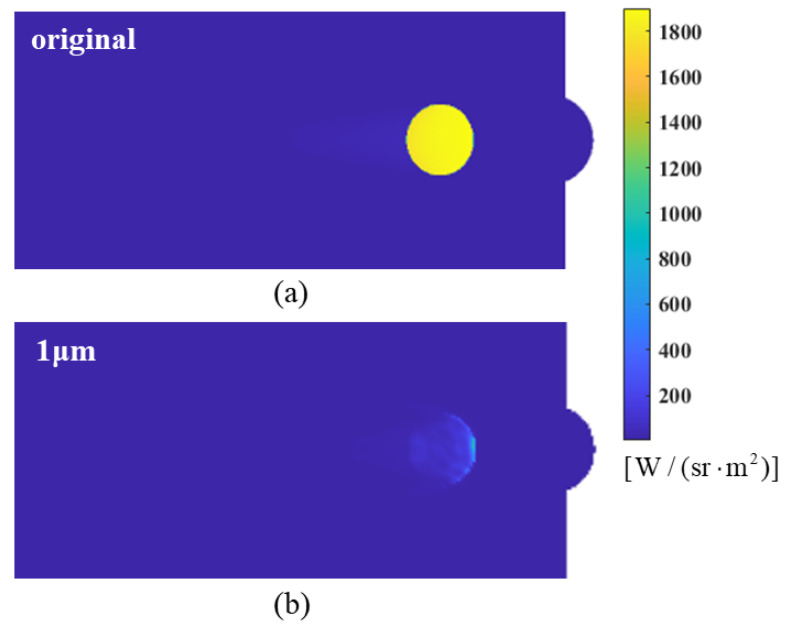
The near-field radiation imaging of the nozzle exhaust system at a detection angle of *ϕ* = 20°: (**a**) original model without aerosol spraying; (**b**) the nozzle sprays aerosol particles with a diameter of 1 µm.

The calculated results of the near-field simulation imaging at *ϕ* = 0° and *ϕ* = 20° are shown in Figure 12 and Figure 13. The figure compares the calculated results of particle injection with a diameter of 1 μm and the original model. It can be seen that the injection of particles decreases the brightness of the spectral radiation, and the high-temperature region of the original model at this wavelength has been obscured. Figure 12 provides the picture after adjusting the color scale to see the less bright areas. The radiance distribution of the particle-injected model exhibits a ‘foggy’ distribution, with a relatively bright region located at the center of the nozzle outlet. The statement suggests that the particle-bearing medium attenuates and scatters the infrared radiation from the exhaust system, resulting in only a small amount of energy being detected at this detection angle.

### 3.3. Influence of Aerosol Mass Flux on Infrared Radiation Intensity

The effect of particle jet mass flux on the infrared radiation signature is mainly through changes in the particle number concentration. At a constant particle size, an increase in the sprayed mass flux increases the particle number concentration at each node in the flow field, thus affecting the radiative transfer. Considering the size of the computational model and the economic efficiency, this paper selects five kinds of aerosol mass fluxes of 0.02 kg/s, 0.05 kg/s, 0.08 kg/s, 0.1 kg/s, and 0.2 kg/s to study the effect of the particle injection flow rate on the infrared radiation of the exhaust system. The particle material is graphite, and the particle size is 1 μm.

**Figure 14:**
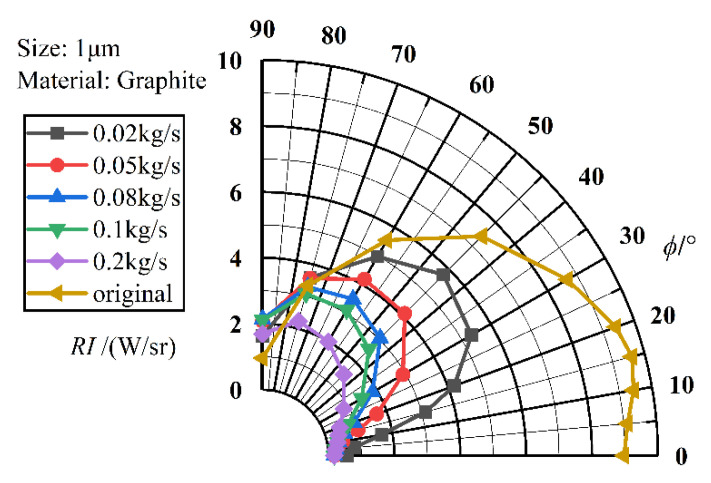
IR intensities under different aerosol mass fluxes.

Figure 14 shows the angular distribution curves of infrared radiation intensity for exhaust systems with different aerosol mass fluxes. Compared to the original model of the axisymmetric exhaust system without particle injection, the computational models at all five flow rates effectively suppress the IR radiation intensity of the exhaust system. The intensity of the infrared radiation decreases as the mass flow rate increases. Each model reaches a minimum at *ϕ* = 0°, increases as *ϕ* increases, reaches a maximum at *ϕ* = 50°–60°, and then decreases.

**Figure 15:**
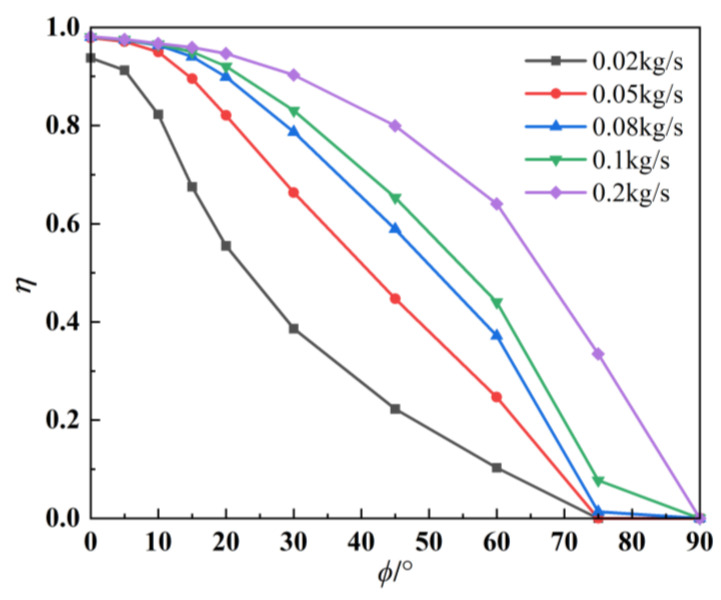
IR suppression rates under different aerosol mass fluxes.

Figure 15 compares the infrared radiation intensity suppression efficiency. Various computational models indicate that the suppression efficiency is highest at *ϕ* = 0°, exceeding 98%. As the detection angle increases, the suppression efficiency gradually decreases and may even result in gain. The aerosol mass flux has a significant impact on the IR suppression effect. At the same detection angle, the IR suppression rate increases with the mass flux. The inhibition rate gradually increases when the mass flux increases from 0.02 kg/s to 0.2 kg/s. However, the increase in the inhibition rate is smaller than the increase in the mass flux. Therefore, it is necessary to choose a suitable aerosol mass flux to achieve a certain inhibition effect without carrying a large amount of aerosol. This paper suggests choosing a mass flux of 0.08 kg/s. When the flux of injected particles reaches 0.08 kg/s, the suppression efficiency is suitable across a wide range of detection angles.

**Figure 16:**
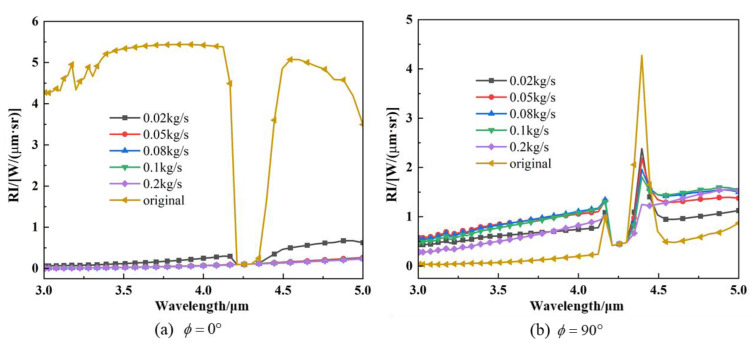
Spectral IR intensities of nozzle exhaust plumes with different particle mass fluxes: (**a**) detection angle of *ϕ* = 0°; (**b**) detection angle of *ϕ* = 90°.

The intensity of spectral radiation at *ϕ* = 0°and *ϕ* = 90° is indicated in the calculations of Figure 16. The spectral radiant intensities of the computational models with different aerosol mass fluxes are generally lower than those of the original model at *ϕ* = 0°. Additionally, there is a significant difference between the models with and without particle injection. Particle injection results in a significant reduction in the radiation intensity, because wall radiation reaches the detector through the medium containing particles at *ϕ* = 0°, and these particles scatter the radiation so strongly that only a small amount of wall radiation reaches the detector. When *ϕ* = 90°, the radiation from the high-temperature wall inside the nozzle is received, the scattering effect of the particle system is evident. The attenuation of the radiant energy is not significant due to the thin shielding layer formed at the nozzle exit. The detector receives more radiant energy than the original calculation model in both instances.

**Figure 17:**
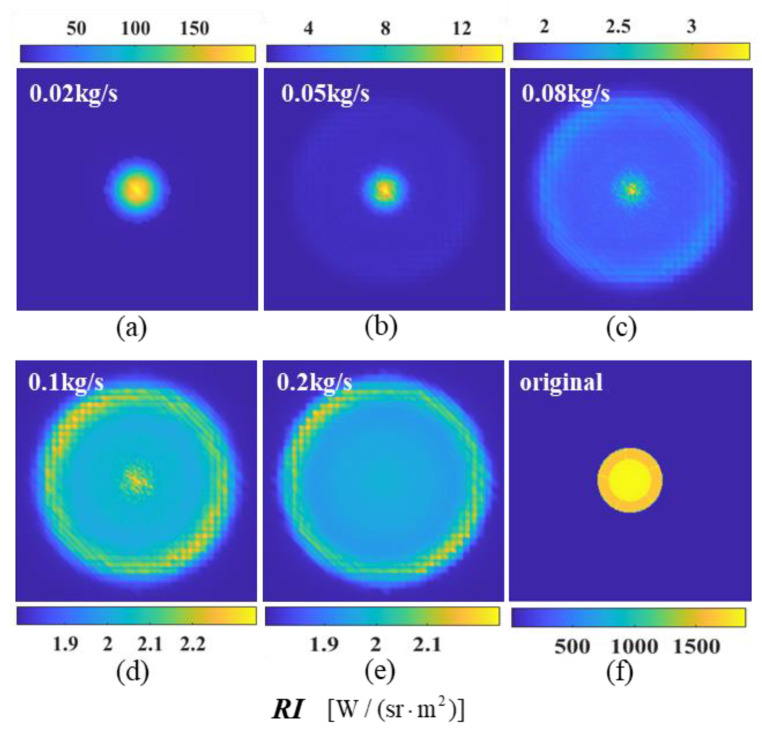
The near-field radiation imaging of the nozzle exhaust system with different particle mass fluxes at a detection angle of *ϕ* = 0°: (**a**) *q_m_* = 0.02 kg/s; (**b**) *q_m_* = 0.05 kg/s; (**c**) *q_m_* = 0.08 kg/s; (**d**) *q_m_* = 0.1 kg/s; (**e**) *q_m_* = 0.2 kg/s; (**f**) *q_m_* = 0.

Figure 17 shows the calculated results of the near-field simulation imaging of six cases at a 0° detection angle. The spectral radiant brightness of each model significantly decreases after particle injection at a 0° detection angle, with an IR suppression efficiency of approximately 98%. As the aerosol mass flux increases, the suppression effect improves, and a slightly higher intensity appears in the outer ring due to the high concentration of aerosol particles. This paper selects a 0.08 kg/s aerosol mass flux based on its inhibition effect and economic benefits.

### 3.4. Influence of Aerosol Materials on Infrared Radiation Intensity

The influence of aerosol materials on the infrared radiation characteristics of thermal jets is mainly reflected in the following aspects:

(1) Changes in the particle densities of different materials can affect the concentration of particle numbers in the flow field, which, in turn, can alter the intensity of infrared radiation, assuming a constant jet mass and particle size.

(2) Assuming a constant particle size and incident wavelength, any variation in the complex refractive index of different particle materials will directly impact the attenuation coefficient and, consequently, the final intensity of infrared radiation.

**Figure 18:**
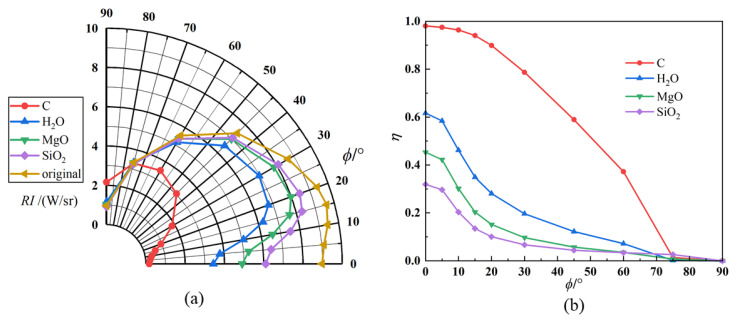
IR characteristics with different particle materials: (**a**) IR intensity of nozzle exhaust plume; (**b**) IR suppression rate.

Figure 18 compares the angular distribution of the infrared radiation intensity and IR suppression rate for exhaust systems with different aerosol particle materials. Compared with the axisymmetric exhaust system without particle injection (original model), all four computational models can effectively suppress the IR radiation intensity of the exhaust system. However, beyond the detection range of 75°, the material models’ IR intensities were even higher than that of the original calculation model. The previous discussion supports that various calculation models achieve maximum suppression efficiency at a 0° detection angle, generally above 80%. As the detection angle increases, the suppression efficiency gradually decreases. Graphite has good suppression efficiencies over a wide range of detection angles, followed by H_2_O, MgO, and SiO_2_.

**Figure 19:**
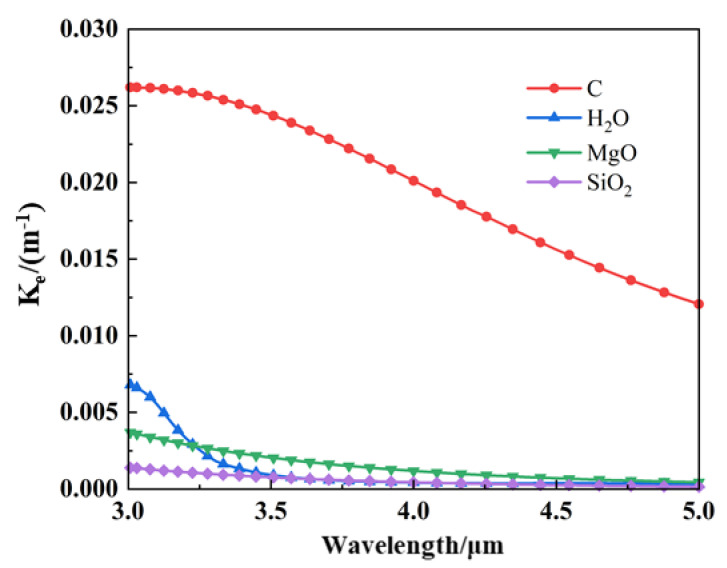
Attenuation coefficients of different aerosol particle materials.

Due to the different complex refractive indices of the particle materials, the attenuation coefficients $\kappa_{e}$ of the particle populations are different for certain particle sizes and incident wavelengths, as shown in Figure 19, resulting in a change in the transmittance of the unit cell when calculating the radiative transfer (see Section 2.3.2). C has a more significant attenuation coefficient and a smaller transmittance and, therefore, a better suppression over a broader range of detection angles.

**Figure 20:**
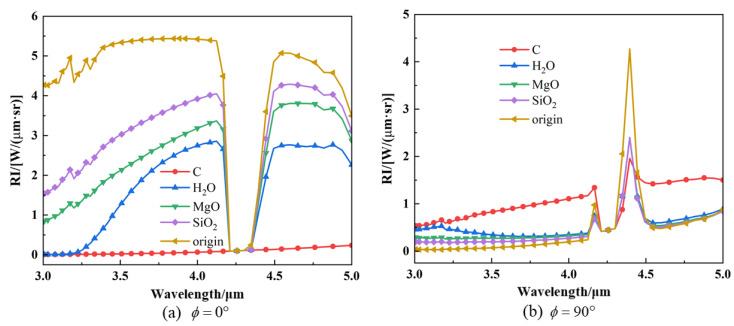
Spectral IR intensities of nozzle exhaust plume with different particle materials: (**a**) detection angle of *ϕ* = 0°; (**b**) detection angle of at *ϕ* = 90°.

The spectral radiation intensities at *ϕ* = 0° and 90° are indicated in the calculations of Figure 20. As illustrated in Figure 20a, the spectral radiation intensities of various aerosol materials are typically lower than that of the original model at *ϕ* = 0°. Significant differences are observed between the various materials, with the graphite material exhibiting the lowest spectral infrared radiation intensity. Furthermore, there is a pronounced decline in the 4.20–4.35 μm band, which is a strong absorption–emission band of CO_2_. Given that the path of radiant energy to reach the detector is relatively long at this angle, it can be seen that the radiant energy in the spectral band from the exhaust system is absorbed by the CO_2_. As illustrated in Figure 20b, at *ϕ* = 90°, the high-temperature solid wall of the nozzle is almost invisible, and the radiation intensity at this detection angle mainly comes from high-temperature gas. However, upon aerosol injection, the solid particle contributes to the overall radiation intensity, and the radiation intensity of the solid is much higher than that of the gas at the same temperature. Different particles at this angle will increase the radiation characteristics of the tail-jet flame; this is primarily due to the particles being capable of scattering the energy, thereby allowing the radiant energy from the solids to reach the detector after multiple scattering events. The 4.20–4.35 μm band exhibits spectral radiation characteristics similar to *ϕ* = 0°, also due to the strong absorption of CO_2_ molecules in the atmosphere. The peak of the curve at the 4.35–4.5 μm band is primarily attributed to the absorption and emission of each component of the tail jet. Furthermore, the aerosol particle exerts a certain suppression effect on the radiation of high-temperature gas at 4.35–4.5 μm band while exhibiting a certain gain for other spectral regions.

**Figure 21:**
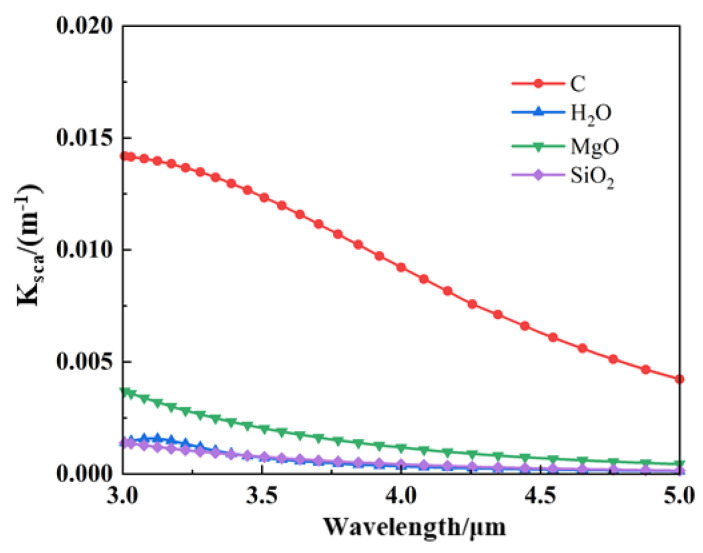
Scattering coefficients of different aerosol particle materials.

**Figure 22:**
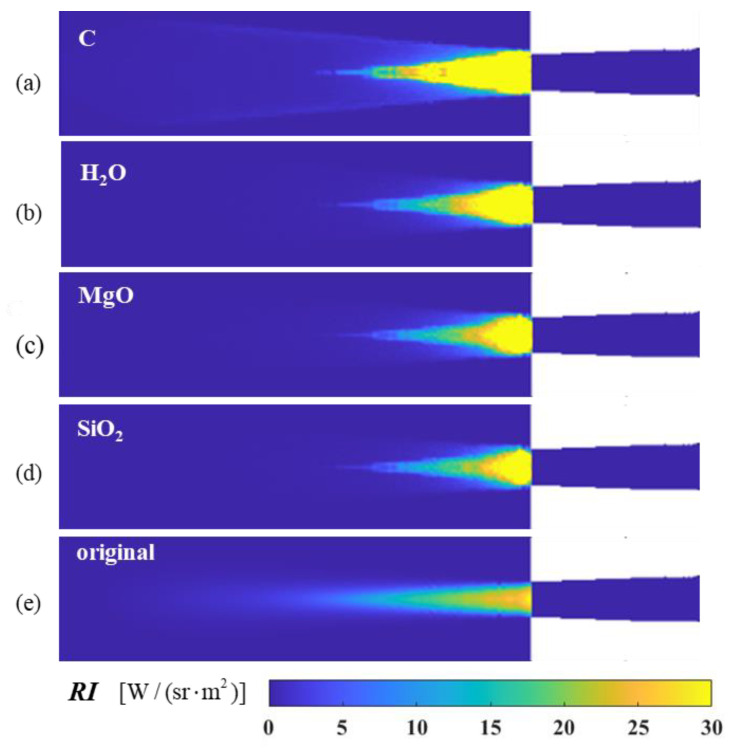
The near-field radiation imaging of the nozzle exhaust system spraying different particle materials at a detection angle of *ϕ* = 90°: (**a**) graphite (C); (**b**) water (H_2_O); (**c**) magnesium oxide (MgO); (**d**) silicon oxide (SiO_2_); (**e**) original model without aerosol spraying.

The flow field with particle injection creates a particle-containing region outside the high-temperature gas wake region, predominantly found in low-temperature environments. Due to the low temperature, the absorption–emission effect of the shield formed by the particles is insignificant. In contrast, the scattering effect is significant, so the radiant energy entering this region is mainly affected by scattering. Additionally, some particles emit energy as they enter the hot gas region. It is important to note that each material has a gain for infrared radiation at *ϕ* = 90°. Figure 21 shows the scattering coefficients $K_{sca}$ for different materials. It can be observed that graphite exhibits a higher scattering coefficient than the other three materials, which can also be attributed to the higher IR intensity of injected graphite than the other materials at *ϕ* = 90°, as illustrated in Figure 18. It can also be seen from the near-field simulation imaging at *ϕ* = 90° in Figure 22 that the injection of aerosol particles enhances the IR intensity to some extent. Nevertheless, in general, the IR intensity at this angle remains below that observed at all other angles.

**Figure 23:**
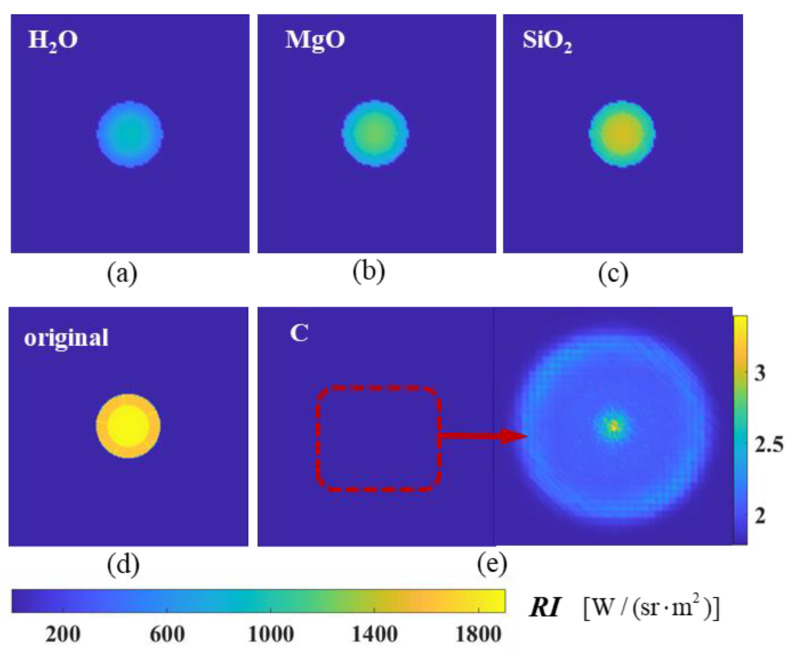
The near-field radiation imaging of the nozzle exhaust system spraying different particle materials at a detection angle of ϕ = 0°: (**a**) water (H_2_O); (**b**) magnesium oxide (MgO); (**c**) silicon oxide (SiO_2_); (**d**) original model without aerosol spraying; (**e**) graphite (C).

The calculated results of the near-field simulated imaging at a 0° detection angle are shown in Figure 23. The near-field imaging results graphically demonstrate the infrared characteristics of the exhaust system components. The figure displays the calculated results of the infrared characteristics directly behind the exhaust system of the convergent nozzle, affected by high-temperature thermal radiation and the attenuation of high-temperature gases on the inner wall surface of the tailpipe. It can be seen from the figure that the spectral radiance of each model after the injection of graphite, H_2_O, MgO, and SiO_2_ particles is smaller than that of the original model at a 0° detection angle. Among them, graphite has the best suppression effect.

From the above analyses, it is clear that graphite particles have a wider range and higher efficiency of IR suppression as an aerosol material among these four materials. Therefore, graphite is used for subsequent studies.

### 3.5. Effect of Nozzle Shape on Infrared Radiation Intensity

This paper uses the typical convergent nozzle, S-bend nozzle, and convergent–divergent nozzle as examples for the research. The flow field settings are the same as those of the convergent nozzle. The nozzle areas of the three types are always kept equal during the design process to avoid the influence of the nozzle area on the flow field.

**Figure 24:**
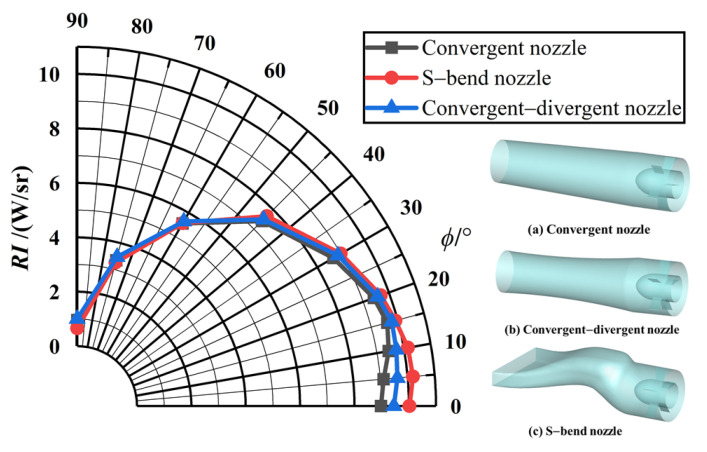
IR intensity of the original exhaust system for different nozzle models.

According to the discussion in Section 3.2, Section 3.3 and Section 3.4, the aerosol particle-spreading condition for all three nozzles is 1 μm graphite, and the injection aerosol mass flux is 0.08 kg/s. As can be seen from Figure 24, the intensity of the raw IR radiation when no aerosol is injected is essentially the same for all three nozzles. There is a slight difference when *ϕ* = 0°, with the S-bend nozzle being high, the convergent–divergent nozzle being second, and the convergent nozzle being low, due to the dominant role of solid-wall radiation. The geometry of the S-bend nozzle results in a disc cavity structure that can be observed, leading to higher IR radiation. The convergent–divergent nozzle has a slightly higher intensity of IR radiation than the convergent nozzle, as the inner wall of the contracting section of the expanding nozzle can also be observed. However, the exit area is the same. As the detection angle increases, the observable solid-wall area decreases, and the infrared radiation gradually decreases.

**Figure 25:**
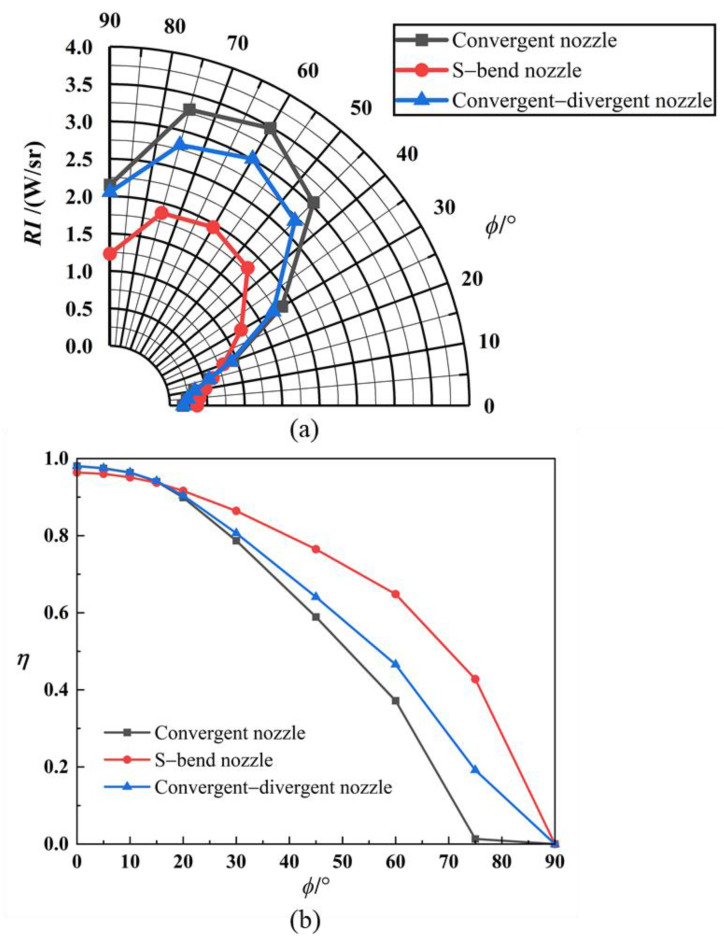
IR characteristics for different nozzle models with particle injection: (**a**) IR intensity of nozzle exhaust plume; (**b**) IR suppression rate.

The characteristics of infrared radiation from the three nozzles are significantly different after the aerosol was sprayed, which is illustrated in Figure 25. The minimum value of IR intensity of nozzle exhaust plume is reached at 0°, and as the detection angle increases, the infrared radiation also increases to some extent, reaching the maximum value at 60°. This is due to the combined effect of tail-flame radiation and solid-wall radiation at this angle. Infrared radiation decreases after 60°. Under the same aerosol dispersal conditions, the suppression of infrared radiation intensity also varies between nozzle shapes (shown in Figure 25b). The suppression efficiency decreases as the detection field of view increases, dropping to zero at *ϕ* = 90°, which is consistent with the phenomenon described in the previous section. The S-bend nozzle has a reasonable suppression rate over a wide range of field-of-view angles, followed by the convergent–divergent nozzle and convergent nozzle.

**Figure 26:**
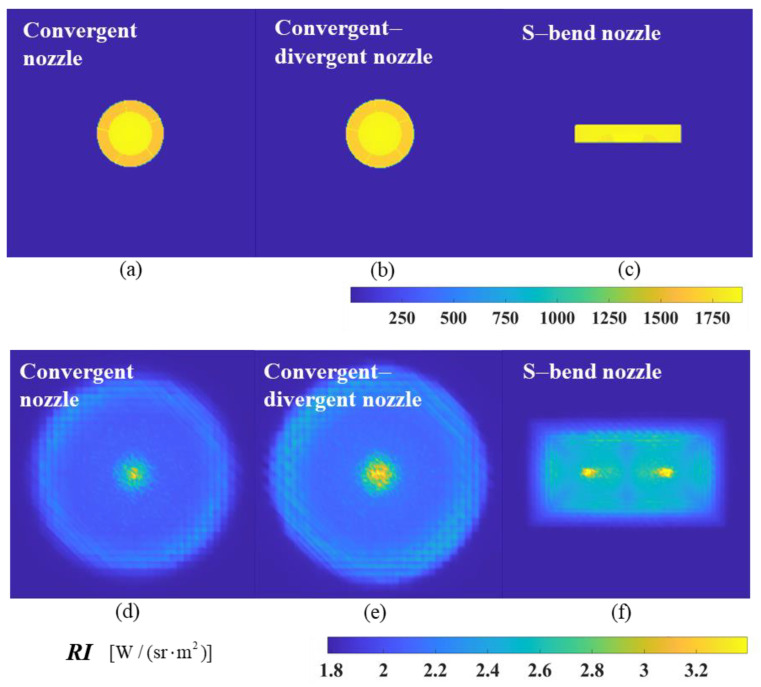
The near-field radiation imaging of different nozzle exhaust systems at a detection angle of *ϕ* = 0°. (**a**) Original model of convergent nozzle without aerosol spraying; (**b**) original model of convergent–divergent nozzle without aerosol spraying; (**c**) original model of S-bend nozzle without aerosol spraying; (**d**) the convergent nozzle sprays graphite particles; (**e**) the convergent–divergent nozzle sprays graphite particles; (**f**) the S-bend nozzle sprays graphite particles.

Figure 26a–c show the calculated results of the near-field simulation imaging of the original model at a 0° detection angle, in which the infrared features of the components inside the nozzle are visible, including the support plate, the inner cone, and the disc cavity structure. After the aerosol injection, the infrared radiation is significantly reduced, showing a ‘foggy’ radiation distribution with a relatively bright area in the center of the nozzle outlet, as shown in Figure 26d–f.

**Figure 27:**
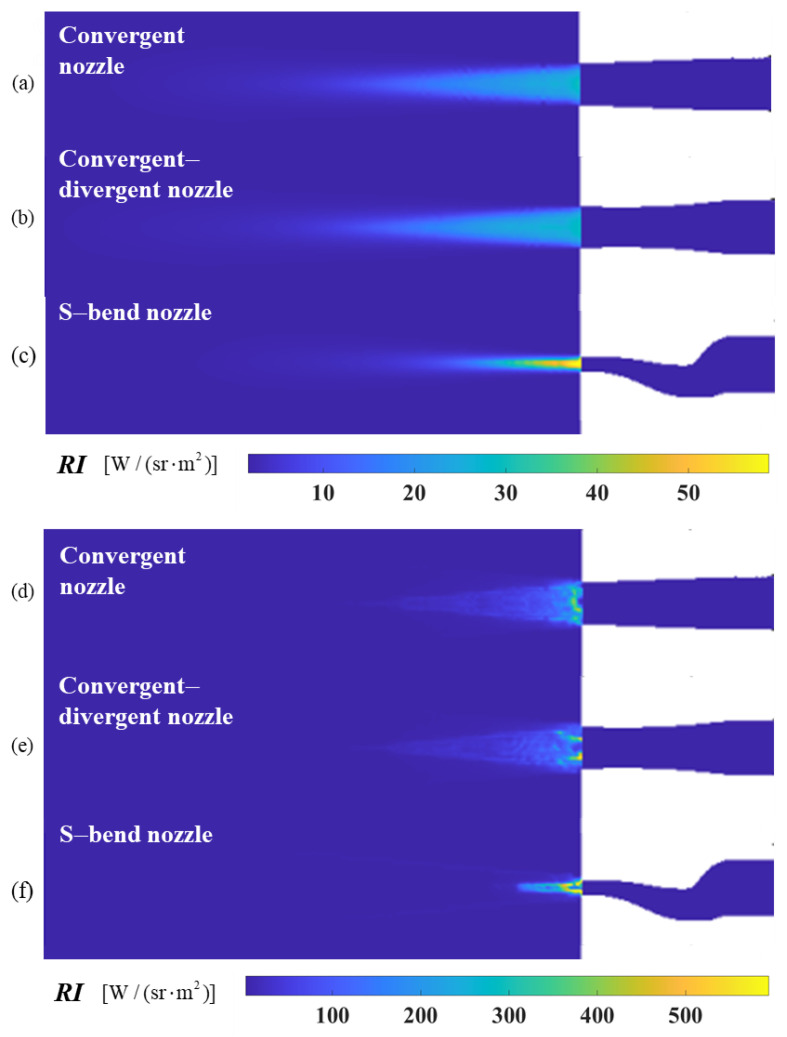
The near-field radiation imaging of different nozzle exhaust systems at a detection angle of *ϕ* = 90°. (**a**) Original model of convergent nozzle without aerosol spraying; (**b**) original model of convergent–divergent nozzle without aerosol spraying; (**c**) original model of S-bend nozzle without aerosol spraying; (**d**) the convergent nozzle sprays graphite particles; (**e**) the convergent–divergent nozzle sprays graphite particles; (**f**) the S-bend nozzle sprays graphite particles.

According to the previous section, at *ϕ* = 90°, the intensity of the infrared radiation increases slightly compared to the original model due to particle scattering, as illustrated in Figure 27. For all three selected nozzle models, the intensity of IR radiation at a 90° detection angle is enhanced after aerosol particles are injected. However, the discrete aerosol particles exhibit the most significant suppression in the range of angles where the flow field radiation intensity is highest (0° to 20°), which is favorable for the overall effect of IR suppression.

Compared to the ordinary axisymmetric convergent nozzle and convergent–divergent nozzle, the S-bend nozzle increases the contact area between the tail jet and the cold air, which promotes the diffusion of aerosol particles and facilitates heat dissipation and gas stream mixing. Simultaneously, it enhances the energy and mass exchange between the high-temperature exhaust and the cold surrounding air, significantly lowering the temperature of the nozzle tail flame and enhancing the aircraft’s infrared stealth capability.

## 4. Conclusions

The influencing factors and laws of aerosol particles on the infrared radiation properties of exhaust plumes are simulated in this study with a model that covers the flow field of the engine nozzle exhaust plumes containing aerosol particles. An automatic preprocessing model is constructed to quickly identify the flow field information. Based on the high-spectral-resolution Planck-weighted narrow spectral band gas model and the RMC method, the infrared radiation characteristics of the exhaust system in the 3–5 µm band range are investigated. The effects of the aerosol material, particle size, mass flux, and nozzle shape are systematically studied. It is demonstrated that the injection of suitable aerosol particles can effectively reduce the intensity of the infrared radiation emitted from exhaust nozzles. The main conclusions of this study are as follows:

(1) The particle size and particle mass flux have a large influence on the IR radiation suppression effect. For a given mass flow rate, the size of the particles determines the particle number density in the flow field. The extinction parameters of the particles also vary with the particle size. These factors are of significant importance in radiative transfer. The suppression effect is less pronounced when the particle size is either small (<1 μm) or large (>5 μm). When the aerosol particle diameter is the same, the higher the particle mass flux, the stronger the radiation suppression ability, and it is necessary to consider the cost and economic efficiency to select the appropriate mass flux. In this study, 1 μm particles with a mass flow rate of 0.08 kg/s have the best suppression effect.

(2) Different aerosol particle materials have different IR radiation suppression efficiencies due to the different complex refractive indices of the particle materials, resulting in further attenuation efficiencies. The most effective suppression effect is achieved when the particle material is graphite, followed by H_2_O and MgO, while SiO_2_ results in the least effective suppression.

(3) There is minimal variation in the suppression of infrared radiation between the three original nozzle models. Under the same aerosol diffusion conditions (graphite, 1 μm, 0.08 kg/s), different shapes of nozzles have different suppression strengths of IR radiation. The S-bend nozzle has the best suppression effect on IR radiation, followed by the convergent–divergent nozzle, and the convergent nozzle has the worst suppression effect.

This study provides guidance for the selection of aerosol and nozzle models for infrared stealth technology. At present, the primary focus is on the radiation suppression effect of aerosol particles on the exhaust system in the ground state. However, various parameters of the vehicle in practical engineering applications, such as the Mach number and flight altitude in the high-altitude cruise state, will also have an impact on the suppression effect. How to realize ultra-long-range detection also needs further research. Furthermore, the numerical model studied in this paper only contains a simple nozzle structure and does not consider the complex structures inside the actual nozzle. The walls of the nozzle are currently treated as a grey medium, whereas the emissivity of the actual nozzle material is not only temperature dependent, but also has significant directional and spectral properties. The next stage of the project will be to further optimize the computational speed of infrared radiative transfer. In addition, experimental studies will be conducted with the objective of more accurately assessing the accuracy of the program or software and providing guidance for engineering calculations.

## Figures and Tables

**Table 1:** Review of previous works on the selected aerosol particle parameters.

Author(s) (Year)	Aerosol Materials	Aerosol Particle Size (µm)	Aerosol Mass Flux (kg/s)
Zhang (2009) [21]	Brass (Cu), nickel (Ni), graphite (C), and asbestos	0.01–100	0.024–1.2
Wang J (2008) [22]	Silicon dioxide (SiO_2_), graphite (C), magnesium oxide (MgO), and aluminum (Al)	0.5–10	0.0216–0.54
Myong (2022) [23]	Graphite (C) and water (H_2_O)	5, 10	15.2, 1.025
Gao (2016) [24]	Silicon dioxide (SiO_2_)	0.1–10	0.04–1

## Data Availability

Data sharing not applicable.

## References

[1] Wang J., Gao J.B., Wang W.N., Wang J.L., Xie X. (2004). Aircraft plume signature suppression and stealth. Proceedings of the Photonics Asia 2004.

[2] Razeghi M., Tournié E., Brown G.J., McEwan K. Military applications for high-performance thermal imaging. Proceedings of the Quantum Sensing and Nanophotonic Devices XII.

[3] Wen S., Zhang Y., Ma Y., Sun Z. (2023). Dirac semimetal-assisted near-field radiative thermal rectifier and thermostat based on phase transition of vanadium dioxide. Opt. Express.

[4] Lu J., Guina M., Gong H., Liu D., Huang H., Wang C., Li S. (2018). Infrared imaging guidance missile’s target recognition simulation based on air-to-air combat. Optical Sensing and Imaging Technologies and Applications.

[5] Fan J.X., Wang F. Analysis of the development of missile-borne IR imaging detecting technologies. Proceedings of the Electro-Optical and Infrared Systems: Technology and Applications XIV.

[6] Paterson J. (1999). Overview of Low Observable Technology and Its Effects on Combat Aircraft Survivability. J. Aircr..

[7] Marcus C., Andersson K., Åkerlind C. (2017). Balancing the radar and long wavelength infrared signature properties in concept analysis of combat aircraft—A proof of concept. Aerosp. Sci. Technol..

[8] Pan Y., Huang L., Sun W., Gao D., Li L. (2021). Invisibility Cloak Technology of Anti-Infrared Detection Materials Prepared Using CoGaZnSe Multilayer Nanofilms. ACS Appl. Mater. Interfaces.

[9] Huang S., Fan Q., Xu C., Wang B., Wang J., Yang B., Tian C., Meng Z. (2020). A visible-light-transparent camouflage-compatible flexible metasurface for infrared–radar stealth applications. J. Phys. D Appl. Phys..

[10] Zhou Z., Huang J. (2021). Mixed design of radar/infrared stealth for advanced fighter intake and exhaust system. Aerosp. Sci. Technol..

[11] Yang Z., Zhang J., Shan Y. (2022). Effects of forward-flight speed on plume flow and infrared radiation of IRS-integrating helicopter. Chin. J. Aeronaut..

[12] Shan Y., Zhang J.Z., Pan C.X. (2013). Numerical and experimental investigation of infrared radiation characteristics of a turbofan engine exhaust system with film cooling central body. Aerosp. Sci. Technol..

[13] Zhang J., Pan C., Shan Y. (2014). Progress in helicopter infrared signature suppression. Chin. J. Aeronaut..

[14] Hu J., Hu Y., Ye Y., Shen R. (2023). Unique applications of carbon materials in infrared stealth: A review. Chem. Eng. J..

[15] Liu X., Liu Y., Peng Q., Jie F., Ming D. Calculation of Attenuation of Infrared Radiation Energy By Ship Water Mist. Proceedings of the 2019 IEEE 8th Joint International Information Technology and Artificial Intelligence Conference (ITAIC).

[16] Qu H.M., Li R., Zhao S.J., Li J.J., Da J., Chen Q. Evaluation of Infrared Stealth Effect Based on Vega Simulation. Proceedings of the 2015 IEEE International Conference on Information and Automation.

[17] Wang F., Liu J., Liu L., Xu L., Wang Y., Chen M., Wang Y. (2021). Quantitative non-destructive evaluation of CFRP delamination defect using laser induced chirp-pulsed radar photothermal tomography. Opt. Lasers Eng..

[18] Shi J., Wang J., Gao Y. Computation of infrared extinction of the brass aerosol. Proceedings of the 25th International Conference on Infrared and Millimeter Waves (Cat. No.00EX442).

[19] Yurkin M.A., Hoekstra A.G. (2016). Corrigendum to “The discrete dipole approximation: An overview and recent developments” [J. Quant. Spectrosc. Radiat. Transfer 106 (2007) 558–589]. J. Quant. Spectrosc. Radiat. Transf..

[20] Wang H., Sun H., Song Z., Liu D., Tian T. (2012). Numerical calculation and analysis of transmittance of smoke screen based on Monte Carlo method. Infrared Laser Eng..

[21] Zhang J.Y., Chang H.P., Wang H.Y. (2009). Numerical simulation on infrared radiation suppressant of hot exhaust with the spherical particles. J. Aerosp. Power.

[22] Wang J. (2008). Research on IR Suppressant of Hot Exhaust with the Discrete Particles. Master’s Thesis.

[23] Yu L., Ji-Won L., Chang S., Jae-Won K., Myong R.S. (2022). Particle Layer Effects on Flowfield and Infrared Characteristics of Aircraft Exhaust Plume. J. Aircr..

[24] Gao X. (2016). Investigation on the Infrared Radiation and Radar Scattering Characteristics of Aircraft and Engine. Ph.D. Thesis.

[25] Sun W.-J., Gao Q.-H., Zhang J.-Z., Hu F., Shan Y. (2023). Aerosol infrared stealth technology: Theory and development of infrared suppression and particle dispersion in aircraft plume. Therm. Sci. Eng. Prog..

[26] Sparks L. (1997). Efficient line-by-line calculation of absorption coefficients to high numerical accuracy. J. Quant. Spectrosc. Radiat. Transf..

[27] Hargreaves R.J., Gordon I.E., Rey M., Nikitin A.V., Tyuterev V.G., Kochanov R.V., Rothman L.S. (2020). An Accurate, Extensive, and Practical Line List of Methane for the HITEMP Database. Astrophys. J. Suppl. Ser..

[28] John R., Howell M., Pinar M., Kyle D., Robert S. (2020). Thermal Radiation Heat Transfer.

[29] Wei L., Li G., Song M., Sun S. (2021). Theoretical investigation on inverse identification of spectral properties of paraffin phase change materials based on multi-thickness model. Sol. Energy.

[30] Bohren C.F., Huffman D.R. (2008). Absorption and Scattering of Light by Small Particles.

[31] Hu Q.-L., Huo J.-T., Miao X.-K., Shao M., Li W.-Z., Kang H.-C. (2021). Simulation of false-alarm area of laser guidance based on Mie scattering model. Optoelectron. Lett..

[32] Wriedt T. (2009). Light scattering theories and computer codes. J. Quant. Spectrosc. Radiat. Transf..

[33] Lentz W.J. (1976). Generating Bessel functions in Mie scattering calculations using continued fractions. Appl. Opt..

[34] Dave J.V. (1969). Scattering of Visible Light by Large Water Spheres. Appl. Opt..

[35] Christian W., Phillip M., Robert V., Doğuşcan A., Christiane B., Ute R. (2020). Metasurface Enhanced Sensitized Photon Upconversion: Toward Highly Efficient Low Power Upconversion Applications and Nanoscale E-Field Sensors. Nano Lett..

[36] Floyd J.E., McGrattan K.B., Hostikka S., Baum H.R. (2003). CFD Fire Simulation Using Mixture Fraction Combustion and Finite Volume Radiative Heat Transfer. J. Fire Prot. Eng..

[37] Georgios E., Bryce S. (2018). Geometrical concentration for enhanced up-conversion: A review of recent results in energy and biomedical applications. Opt. Mater..

[38] Doyoung B., Changjin L., Seung W. (2004). Radiative heat transfer in discretely heated irregular geometry with an absorbing, emitting, and anisotropically scattering medium using combined Monte-Carlo and finite volume method. Int. J. Heat Mass Transf..

[39] Lu X., Hsu P.-F. (2005). Reverse Monte Carlo simulations of light pulse propagation in nonhomogeneous media. J. Quant. Spectrosc. Radiat. Transf..

[40] Gao B.-H., Qi H., Yin Y.-M., Wei L.-Y., Ren Y.-T. (2019). Fast reconstructing two-dimensional temperature distribution in participating media with different surfaces conditions. Infrared Phys. Technol..

[41] Gao B.-H., Qi H., Jiang D.-H., Ren Y.-T., He M.-J. (2021). Efficient equation-solving integral equation method based on the radiation distribution factor for calculating radiative transfer in 3D anisotropic scattering medium. J. Quant. Spectrosc. Radiat. Transf..

[42] Gao B.-H., Qi H., Zhao Y., Ren Y.-T., He M.-J. (2021). An efficient equation-solving method for calculating radiative transfer in isotropic scattering medium. Int. J. Heat Mass Transf..

[43] Cui L., Zhang B., Li J.Q., Lin L.B., Ai J.Q. (2021). The Investigation of Flow and Infrared Characteristics of Circle-to-Rectangular C-D Nozzle with a Differently Shaped Convergent Duct. Infrared Technol..

